# Synthesis of BaZrS_3_ Perovskite Thin Films via Different Solid BaS_*x*_ Intermediate Phases

**DOI:** 10.1021/acsomega.6c01800

**Published:** 2026-07-10

**Authors:** Corrado Comparotto, Younes Lablali, Olivier Donzel-Gargand, Tomas Kubart, Francesco Stancari, Jonathan J. S. Scragg

**Affiliations:** † Division of Solar Cell Technology, Department of Materials Science and Engineering, 8097 Uppsala University, Uppsala 75237, Sweden; ‡ Department of Materials Science, Energy and Nano-Engineering, Mohammed VI Polytechnic University, Benguerir 43150, Morocco; § Division of Solid-State Electronics, Department of Electrical Engineering, Uppsala University, Uppsala 75237, Sweden; ∥ Department of Chemistry, Life Sciences and Environmental Sustainability, University of Parma, Parma 43124, Italy

## Abstract

The BaZrS_3_ perovskite has emerged as a promising compound for photovoltaic (PV) applications due to its unique optoelectronic properties, stability, and abundance of the constituent elements. Recent research has devoted considerable effort to decreasing the processing temperature to facilitate the integration of BaZrS_3_ thin films into optoelectronic devices. In this context, the formation of a barium polysulfide liquid flux as an intermediate phase is anticipated to promote BaZrS_3_ synthesis. This study investigates whether solid binaries can also accelerate the process at lower temperatures, i.e., below melting. To address this question, Ba–Zr precursors were sulfurized under various conditions that favor the formation of distinct solid BaS_*x*_ intermediate phases during BaZrS_3_ synthesis, achieved by deliberately selecting processing temperatures below the melting points of any of the involved binary sulfides. The crystallinity of the resulting BaZrS_3_ thin films is evaluated by X-ray diffraction and transmission electron microscopy, revealing that the sulfurization conditions that favor solid BaS_3_ offer a greater advantage for BaZrS_3_ formation than those that promote BaS_2_, which in turn prove more favorable than those that favor BaS. Moreover, by varying sulfur partial pressure and sample temperature during sulfurization, this study disentangles the effects of these two parameters on BaZrS_3_ crystallization: the results indicate that the sulfurization conditions that favor BaS_3_ effectively promote BaZrS_3_ nucleation, while high temperatures predominantly enhance grain growth.

## Graphical abstract

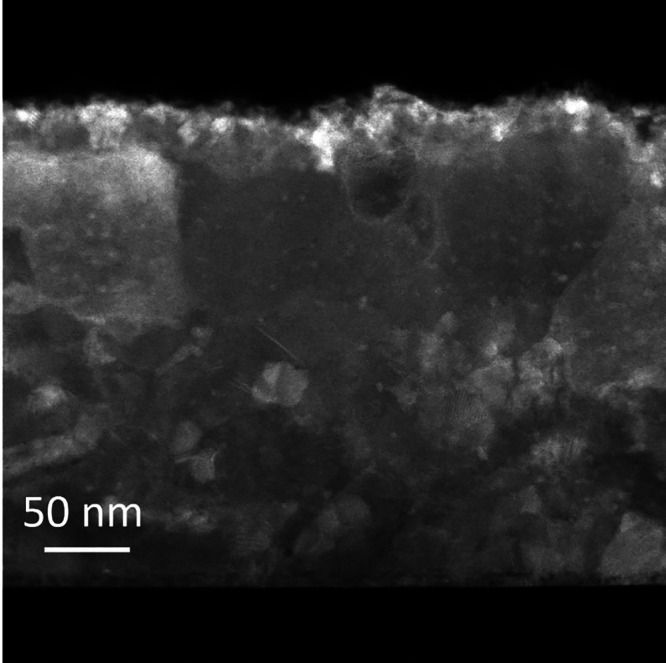

## Introduction

Si-based solar cells have long been the foundation of photovoltaic (PV) technology,^1^ but their power conversion efficiency is steadily approaching the theoretical limit.^2,3^ New absorber materials can be integrated into existing solar cell technologies to improve their performance.^4^ A common approach is to stack two or more subcells, each being more efficient at absorbing a specific part of the light spectrum.^5^ Among the novel materials suited to augment Si-based solar cells, Pb-halide perovskites have shown many remarkable properties, such as strong light absorption,^6^ large diffusion length,^6,7^ high charge carrier mobility,^8^ defect tolerance,^9,10^ ease of fabrication,^11^ and band gap tunability.^12^ However, long-term stability^13^ and the presence of toxic Pb^14^ remain concerns and the PV community has therefore recently begun to look for alternatives. In this context, chalcogenide perovskites have attracted attention due to their optoelectronic properties.^15^ In particular, BaZrS_3_ exhibits a band gap that is almost ideal for a top subcell in a tandem device,^16–18^ and an outstanding absorption has been experimentally measured.^16,18^ Compared to the more common Pb-halide perovskites,^19^ BaZrS_3_ manifests superior thermal^20,21^ and water stability^17^ and is only composed of Earth-abundant, nontoxic elements.^22^ Early synthesis of BaZrS_3_ thin films required high process temperatures,^18,23,24^ complicating its possible integration into a tandem solar cell. Although recent works have partially addressed this issue,^25–28^ films produced at relatively high temperatures (500–600 °C) still exhibit rather small grains, which are believed to contribute to low carrier mobility.^29,30^ Progress toward device integration requires further improvements in material growth without increasing (and ideally by reducing) the growth temperature. Investigations into BaZrS_3_ growth at moderate temperatures show that it seems to be accelerated in S-rich conditions.^31^ This has been related to the formation of a high-order binary polysulfide of Ba (BaS_*x*_ with *x* ≥ 3) that forms as an intermediate phase and acts as a liquid flux,^28,32^ overcoming mass-transport limitations, promoting crystal growth and improving crystallinity. Vincent et al. identified the molten intermediate phase as BaS_*x*_ with *x* > 3, forming at 525 °C.^28^ Yang et al, on the other hand, suggested that liquid BaS_3_ is responsible for the accelerated chemical reaction, which they observed melting at 540 °C.^32^ If a liquid intermediate proves to be a perquisite for effective crystallization, it may impose a lower limit on the synthesis temperature of BaZrS_3_. However, certain solid phases could also provide an accelerating effect. The role of BaS_3_ in low-temperature reactions was explored in a related study, which examined its reaction with another transition metal, Fe, to form BaFe_2_S_3_.^33^ In this case, the authors did not report the involvement of any molten species. Instead, they described the process as a thermally assisted solid-state intercalation, in which Fe gradually diffuses from the surface to the bulk, cleaving S–S bonds in the process. They proposed that the structural transformation of the precursor proceeds with minimal reorganization of its framework, classifying the chemical reaction as topochemical. This low-energy pathway enabled BaFe_2_S_3_ to form at temperatures as low as 340 °C. Interestingly, the authors also observed a similar mechanism in the intercalation of another transition metal, Ni, into BaS_2_ to form BaNiS_2_. This finding suggests that the accelerated reaction at low temperature is associated with the presence of S–S bonds, which are found in both BaS_3_ and BaS_2_ but absent in BaS. Although BaZrS_3_ exhibits a different crystal structure, it would be worthwhile to explore whether a similar solid-state process plays a role in its synthesis at moderate temperatures and to determine the relative effects of BaS_3_ and BaS_2_ compared to BaS in this context. This study addresses these questions by sulfurizing Ba–Zr precursors under conditions aimed at promoting the formation of different solid binary Ba sulfides as intermediate phases while keeping the processing temperature below their melting points. These conditions were selected based on a previous publication that elucidates the ranges of S pressure and temperature required to produce the Ba binaries.^34^ The prepared BaZrS_3_ thin films are analyzed by grazing incidence X-ray diffraction (GI-XRD) and dark-field scanning transmission electron microscopy (DF-STEM), and the observed differences in crystalline quality are eventually correlated with the specific intermediate phases. Finally, BaZrS_3_ nucleation and grain growth are disentangled, separating the effect of the intermediate phase from that of temperature. While the optimization of processing parameters for device-quality films is beyond the scope of this work, the present study aims to provide fundamental insights into the reaction pathways and intermediate phases governing BaZrS_3_ formation. Such understanding is expected to guide future efforts toward the rational design of efficient optoelectronic devices.

## Results and Discussion

### BaZrS_3_ Formation under Selected Conditions

Samples were synthesized using the same sulfurization furnace and method as described in a previous work, in which the regimes of stability of different Ba–S phases were first defined.^34^ The key difference is now the addition of Zr. Briefly, Zr and Ba were cosputter-deposited onto a Si substrate, and an in situ SnS capping layer was grown to mitigate the formation of stable, irreversible oxide products during the subsequent sample transfer and sulfurization. These precursors were then sulfurized in a tube furnace to form BaZrS_3_, with different combinations of S source temperature (*T*_S_) and sample temperature (*T*_sample_). *T*_S_ can be related to the corresponding partial pressure of S (*p*_S_) using the published vapor pressure curve.^35^ To select sulfurization conditions that favor the formation of specific BaS_*x*_ intermediates, the experimental *p*_S_–*T*_sample_ phase diagram for the Ba–S system from the previous work was used.^34^ In Figure 1, the chosen reaction conditions are labeled A–E. Importantly, they are all below the melting temperature of any of the phases concerned. At *T*_sample_ = 500 °C, all three binary phases are accessible in the employed sulfurization furnace, namely, BaS (Experiment C), BaS_2_ (Experiment B), and BaS_3_ (Experiment A). GI-XRD was performed on the sulfurized samples. Figure 2a presents the diffraction patterns of samples sulfurized for 1 min with *T*_sample_ = 500 °C.

**Figure 1:**
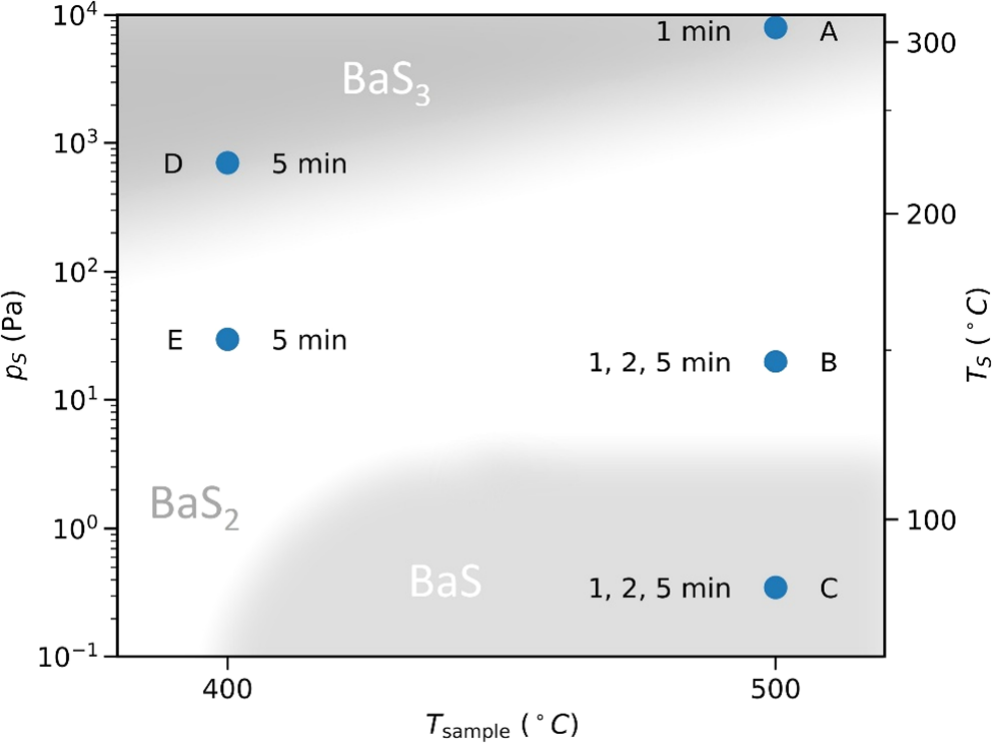
Parameters used in the BaZrS_3_ Formation under Selected Conditions section. Shaded regions indicate the Ba binary phases reported to form under the corresponding processing conditions in the previous experimental work.^34^

**Figure 2:**
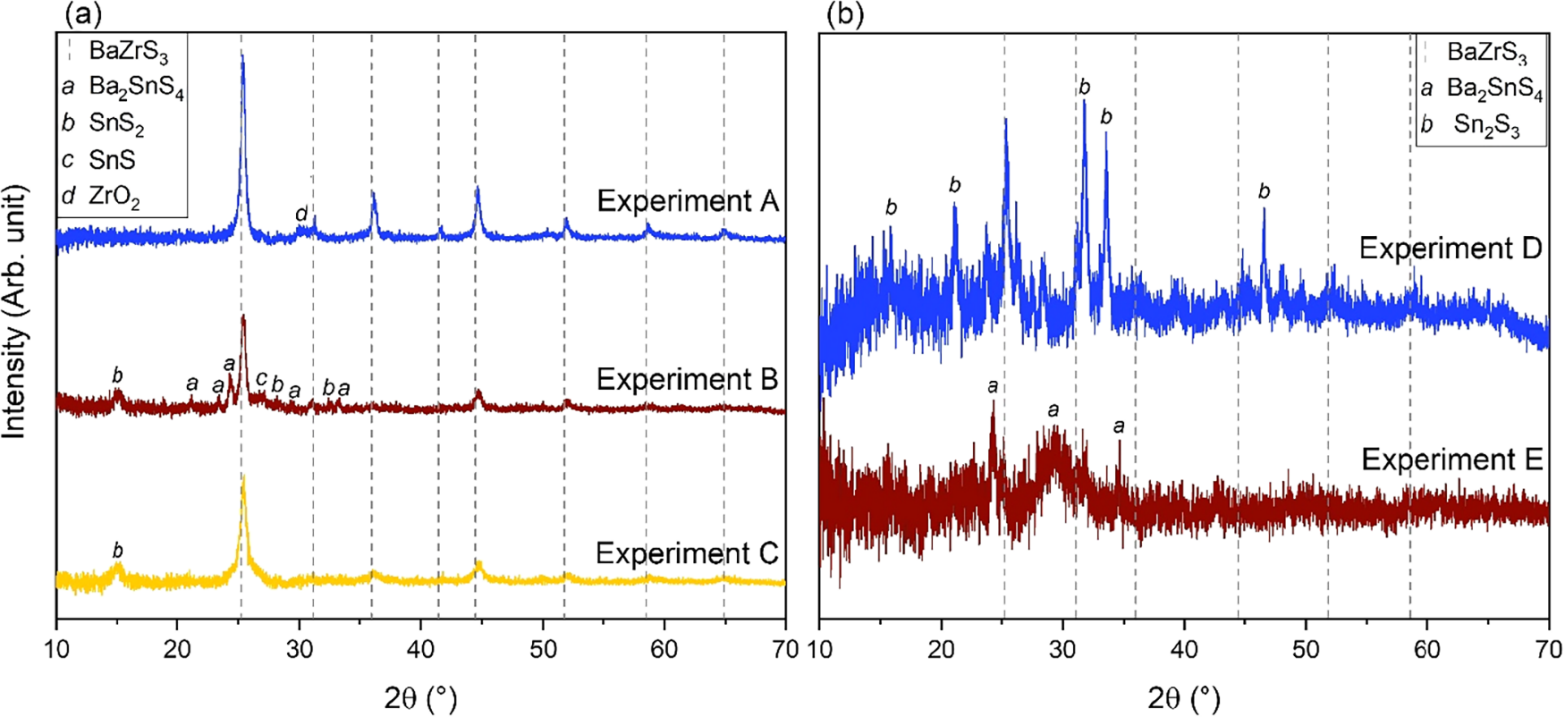
GI-XRD diffraction patterns of samples sulfurized (a) at 500 °C for 1 min and (b) at 400 °C for 5 min. The reference patterns for BaZrS_3_, Ba_2_SnS_4_, Sn_2_S_3_, SnS_2_, SnS, and ZrO_2_ are taken from Lelieveld and Ijdo,^36^ Susa and Steinfink,^37^ Mootz and Puhl,^38^ Pałosz and Salje,^39^ Avellaneda et al.,^40^ and Stocker and Collongues,^41^ respectively.

As can be seen, BaZrS_3_ is present in all three cases, underscoring the rapid chemical reaction at this *T*_sample_. However, the sample from Experiment A clearly exhibits a greater number and higher intensity of BaZrS_3_-related peaks. Giving the same sample size, film thickness, and measurement conditions, the XRD patterns indicate that Experiment A produces the perovskite with the highest degree of crystallinity and/or the largest grain size. This result suggests that the highest *T*_S_ is favorable for BaZrS_3_ formation. Only a broad minor impurity peak at around 30° is detected, which can be attributed to ZrO_2_. In Experiments B and C, peaks belonging to SnS_2_ are observed, probably originating from the reaction between the SnS capping layer and the S-rich atmosphere. In Experiment B, an additional peak attributable to SnS is present. Furthermore, peaks corresponding to Ba_2_SnS_4_ are also observed in Experiment B. The formation of this compound has been reported before.^25^

DF-STEM was performed on these three samples, and the corresponding images are presented in Figure 3. A thin impurity layer is observable at the surface of all three samples, with the thickness varying between approximately 30 and 70 nm. On the basis of the GI-XRD diffraction patterns presented in Figure 2a, this layer is primarily composed of SnS_2_ in experiment C, while experiment B contains both SnS_2_ and Ba_2_SnS_4_ as the dominant crystalline phases. On the other hand, no Sn-containing phases were detected by XRD in experiment A, suggesting that the impurity layer is either amorphous or primarily composed of BaO and/or ZrO_2_. Below this surface layer lies the BaZrS_3_ film, and the evolution of the grain size with *T*_S_ can be clearly observed. At the highest *T*_S_ (Figure 3a), the BaZrS_3_ layer is compact and composed of large grains, particularly in the upper region, with the largest grains reaching several hundred nanometers. As *T*_S_ decreases, the grain size progressively diminishes, and by Figure 3c, the largest grains are reduced to only a few tens of nanometers.

**Figure 3:**
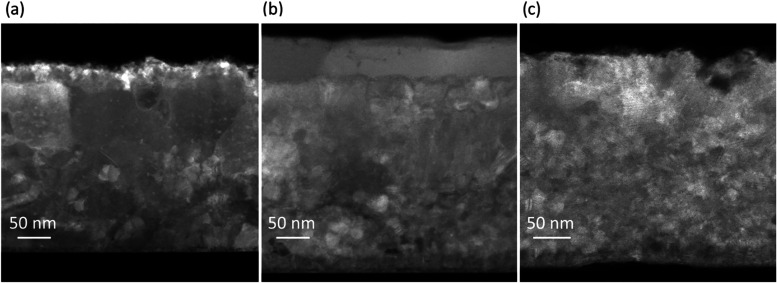
DF-STEM cross-sectional images of BaZrS_3_ samples sulfurized for 1 min from Experiments (a) A, (b) B, and (c) C.

To further compare Experiments B and C, sulfurization durations of 2 and 5 min were also investigated. Figure S1 displays the corresponding GI-XRD patterns, alongside those for 1 min. However, because the high *p*_S_ in experiment A can only be sustained for a short time without depleting the S source in the furnace used in this study, the sulfurization duration was limited to 1 min. The full width at half-maximum (FWHM) of the main (121) BaZrS_3_ diffraction peak of all samples from experiments A, B, and C was calculated, and the corresponding results are presented in Figure 4.

**Figure 4:**
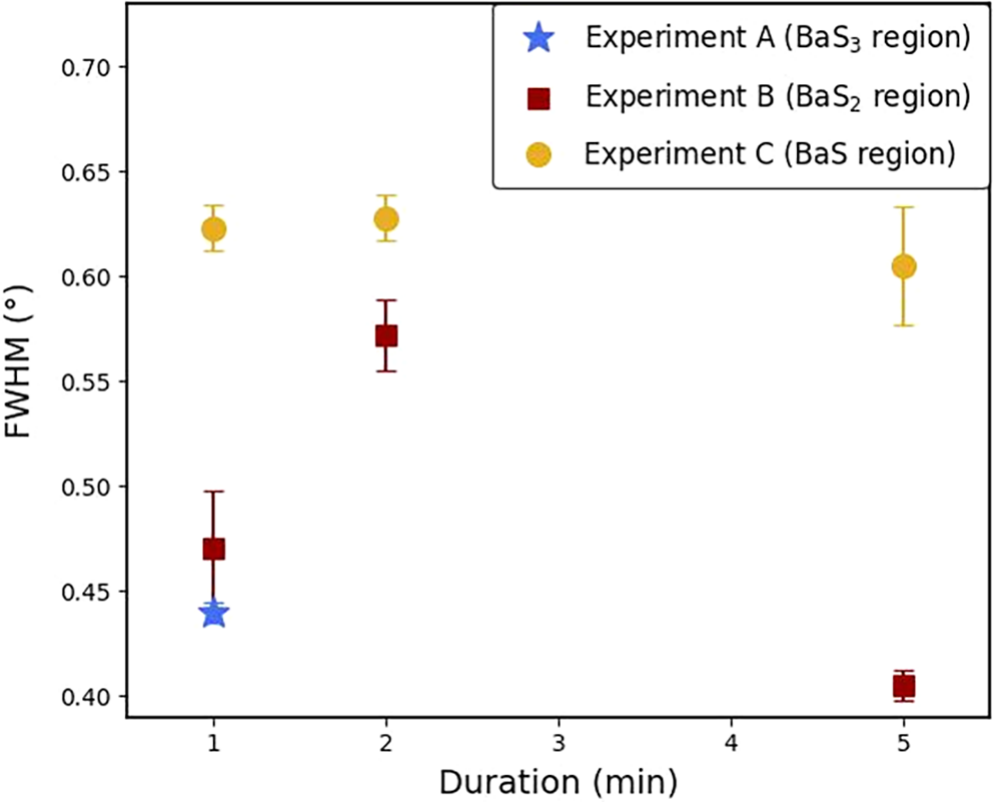
FWHM of the main (121) BaZrS_3_ diffraction peak for the samples sulfurized with *T*_sample_ = 500 °C. The binary sulfides shown in the legend indicate the phases that formed in a previous study under the same sulfurization conditions but in the absence of Zr.^34^ The bars represent the standard error from the fit using a Voigt function in combination with a Levenberg–Marquardt algorithm.

Notably, the FWHM of the films from experiment B is consistently lower than that of the samples from experiment C, regardless of the sulfurization duration. This outcome suggests that a higher *T*_S_ is more advantageous for the formation of BaZrS_3_.

Additionally, the results after a 1 min sulfurization indicate that Experiment A yielded the lowest FWHM, despite some overlap in the error bars from the fits. However, the combined XRD and STEM analyses clearly show that Experiment A produced the highest degree of BaZrS_3_ crystallinity, suggesting that high *T*_S_ facilitates perovskite formation. It should be stressed that this acceleration occurred without the presence of any liquid flux (as opposed to the works by Yang et al.^32^ and Vincent et al.^28^), as the synthesis temperature here was limited to 500 °C in all cases—below the melting point of any binary phase involved.^28,32,42,43^

Regarding the sulfurization duration, its influence on BaZrS_3_ crystallinity appears to be less significant than that of *T*_S_, with no clear trend being observable within the investigated time range. Experiment B with a 2 min sulfurization appears to be an outlier; however, its FWHM remains lower than that of experiment C, not affecting the overall comparison between experiment B and experiment C.

To further investigate the role of *T*_S_ on BaZrS_3_ formation, a reduced *T*_sample_ of 400 °C was also explored in Experiments D and E. In this case, only a sulfurization duration of 5 min was investigated. Figure 2b presents the corresponding GI-XRD diffraction patterns. Experiment E yielded a predominantly amorphous material, with the only discernible peaks—broad and of low intensity—attributable to the impurity Ba_2_SnS_4_, as discussed above. In contrast, Experiment D exhibited several low-intensity peaks attributable to BaZrS_3_, reinforcing the concept that high *T*_S_ facilitates BaZrS_3_ formation. The predominant impurity phase identified in Experiment D is Sn_2_S_3_, unlike SnS_2_ or Ba_2_SnS_4_ observed in the earlier experiments. In addition to the processing conditions *T*_S_ and *T*_sample_, other competing mechanisms may influence which impurity phase forms. For instance, how readily BaZrS_3_ forms can affect the local availability of S vapor for reacting with the SnS capping layer.

All the results so far indicate that a high *T*_S_ is beneficial for BaZrS_3_ formation. As different intermediate Ba binary phases can appear during the formation of the perovskite at different *T*_S_, establishing a correlation between BaZrS_3_ crystalline properties and intermediate binary phases is therefore of considerable scientific interest. In this work, it was attempted to provide direct evidence by interrupting the sulfurization process prior to complete conversion to the perovskite and identifying the resulting phases by GI-XRD. However, no Ba–S binary compounds were detected in any of the experiments, suggesting a rapid chemical reaction. More attempts were then made by placing a dummy sample—consisting of Ba capped with SnS—adjacent to the main sample during the sulfurization process. This approach was intended to form BaZrS_3_ in the main sample and a Ba binary phase in the dummy sample within the same sulfurization run. Under these conditions, however, BaZrS_3_ formation differed from that observed in the absence of the dummy sample. This discrepancy is attributed to the presence of additional SnS, which altered the partial pressure of S. Therefore, establishing a correlation between an intermediate Ba binary phase and the properties of BaZrS_3_ films requires an indirect approach. One such approach is to reference the previously reported experimental *p*–*T* phase diagram of the Ba–S system.^34^ The fabrication conditions employed for BaZrS_3_ in this study can thus be mapped onto the corresponding Ba binary phases identified by the diagram, as indicated in Figure 1.

In this light, individual comparisons in the BaZrS_3_ Formation under Selected Conditions section—i.e., the GI-XRD patterns for samples sulfurized at 400 °C, the GI-XRD patterns for samples sulfurized at 500 °C, the FWHM of the (121) BaZrS_3_ diffraction peak for samples sulfurized at 500 °C, and the DF-STEM cross-sectional images—consistently suggest that the conditions used for the synthesis of the (solid) BaS_3_ intermediate phase promote BaZrS_3_ formation more effectively than those that produce BaS_2_, which in turn are more favorable than those that yield BaS. Since no molten phases are expected to be involved at these temperatures, the beneficial effect associated with the increasing order of the binary Ba polysulfide may be attributed to enhanced solid-state diffusion via intercalation of Zr into BaS_2_ and BaS_3_, similar to the mechanism observed in the related compounds BaFe_2_S_3_ and BaNiS_2_, as discussed in the Introduction.^33^

However, as described previously, a direct relationship between binary Ba intermediate phases and the crystalline properties of BaZrS_3_ has yet to be established, and further investigation is, therefore, required. In situ studies would provide a valuable means of this purpose.

### Cross-Experiments

The previous experiments indicate that an increased *p*_S_ promotes the formation of BaZrS_3_. In addition, *T*_sample_ has a pronounced impact on film crystallinity, as demonstrated by the substantially poorer crystalline quality observed at 400 °C (Figure 2b) compared with 500 °C (Figure 2a). However, the respective roles of *p*_S_ and *T*_sample_ in nucleation and grain growth remain intertwined. To disentangle the effects of these two parameters on BaZrS_3_ formation, both were systematically varied within a single sulfurization run in three additional experiments, under the conditions summarized in Table 1.

**Table 1:** Parameters Used for Cross-Experiments^a^

	Initial 10 min (“nucleation” stage)			Final 10 min (“growth” stage)
Sample name	Expected intermediate phase^a^	Set *T*_S_ (°C)	Set *T*_sample_ (°C)	Set *T*_sample_ (°C)
BaS_3_(−)	BaS_3_	260	400	400
BaS_2_(−)	BaS_2_	260	535	535
BaS_3_(↑)	BaS_3_	260	400	535

a The expected intermediate phases are based on a previous work.^34^

Based on the results obtained in the BaZrS_3_ Formation under Selected Conditions section for the samples sulfurized with a *T*_sample_ of 400 °C (Experiments D and E), poor crystallinity can be expected for some of the conditions in the Cross-Experiments section (BaS_3_(−) and possibly BaS_3_(↑)). In order to favor crystallization, longer times have been chosen here for a fairer comparison within the same set of experiments. Therefore, 20 min processes were divided into two 10 min stages—loosely named “nucleation” and “growth”. In the “nucleation” stage, *T*_S_ and *T*_sample_ were set to target either BaS_2_ or BaS_3_ as the intermediate phase, following the above-discussed indirect approach to reference the process conditions to the previously reported experimental *p*–*T* phase diagram of the Ba–S system.^34^ After 10 min, the S source was rapidly cooled to suppress further sulfurization. The samples then underwent a 10 min “growth” stage in the resulting S-poor atmosphere, during which *T*_sample_ was either maintained (denoted as (−)) or increased (denoted as (↑)). Should the intermediate solid BaS_3_ phase have any positive effect on BaZrS_3_ nucleation, this would be attributable to the “nucleation” stage, as BaS_3_ is not expected to form during the S-poor conditions used for the “growth” stage. On the other hand, should *T*_sample_ have the strongest impact on nucleation, the intermediate phase formed during the “nucleation” stage would play a minor role on the crystallinity of the resulting BaZrS_3_ thin films. The three samples were analyzed using GI-XRD and the resulting diffraction patterns are presented in Figure 5.

**Figure 5:**
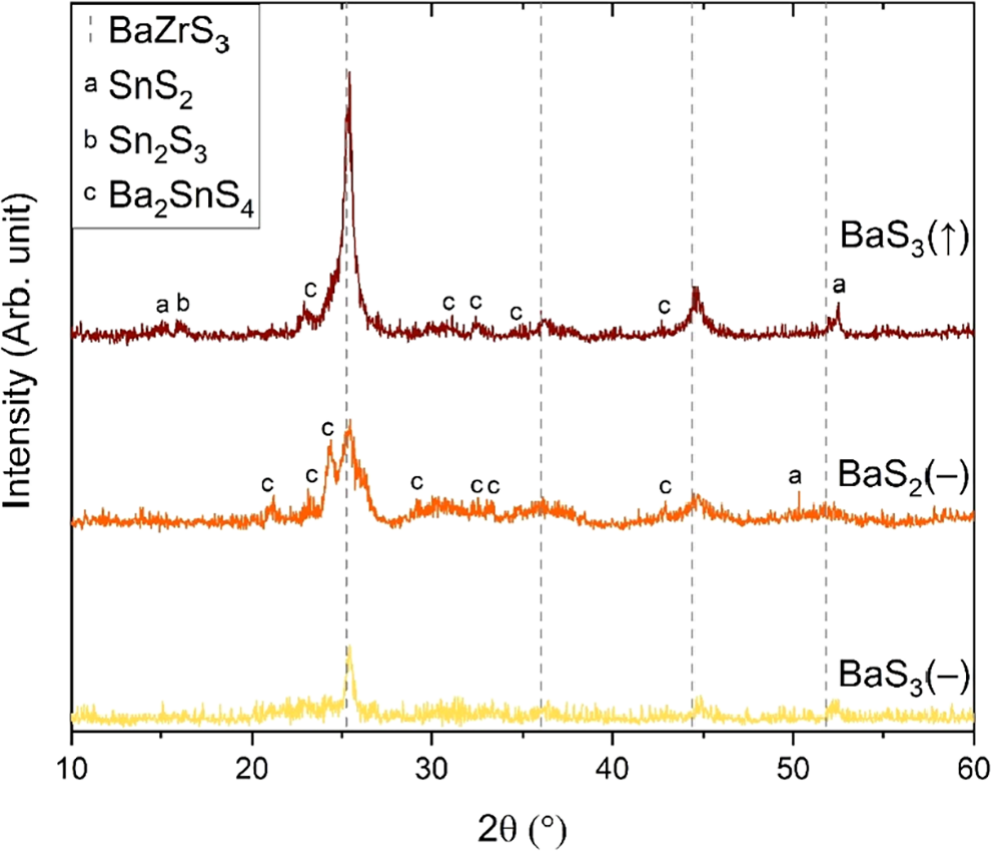
GI-XRD diffraction patterns of the samples synthesized in the cross-experiments. The reference patterns for BaZrS_3_, SnS_2_, Sn_2_S_3_, and Ba_2_SnS_4_ are taken from Lelieveld and Ijdo,^36^ Pałosz and Salje,^39^ Mootz and Puhl,^38^ and Susa and Steinfink,^37^ respectively.

The main impurities detected in the cross-experiments are SnS_2_, Sn_2_S_3_, and Ba_2_SnS_4_, similar to that observed in the BaZrS_3_ Formation under Selected Conditions section. In sample BaS_3_(−), diffraction peaks attributable to BaZrS_3_ are observed, albeit with low intensity. BaZrS_3_ was also detected in sample BaS_2_(−), but the significantly higher *T*_sample_ in both “nucleation” and “growth” stages did not lead to a substantial improvement in crystallinity compared to sample BaS_3_(−). When compared to Experiment B, sample BaS_2_(−) seems to exhibit a lower degree of crystallinity. This may be attributed to the longer sulfurization duration, which likely caused the SnS capping layer to evaporate well before the completion of the process, thereby eliminating its protective effect. Finally, the highest degree of crystallinity was observed in sample BaS_3_(↑), although the total thermal budget provided in this case was lower than that provided to sample BaS_2_(−). This outcome suggests that the formation of BaS_3_ during the “nucleation” stage promoted BaZrS_3_ nucleation and that the higher temperature in the “growth” stage primarily accelerated crystal growth, yielding the most crystalline material among the three experiments.

In conclusion, comparison of sample BaS_3_(↑) with sample BaS_2_(−) indicates that BaS_3_ predominantly influenced nucleation, whereas comparison with sample BaS_3_(−) suggests that *T*_sample_ primarily governed grain growth.

## Experimental Methods

### Sample Preparation

A thin Zr layer (ca. 10 nm) was initially grown onto Si/SiO_*x*_ substrates to prevent Ba reacting with Si during the subsequent sulfurization. The deposition was carried out using a Kurt J. Lesker sputtering system, operating with a base pressure on the order of 10^–5^ Pa. The Zr target—supplied by Plasmaterials, Inc., with a purity of 99.7% excluding Hf—was powered by pulsed direct current (DC) for 4 min with a power density of 1.21 Wcm^–2^. Zr and Ba were then cosputter-deposited in the same chamber for 50 min, the latter being operated in radiofrequency mode. The power densities were 0.81 and 0.86 Wcm^–2^, respectively. The purity of the Ba target was 99.5%. Finally, a SnS capping layer was deposited in situ atop the Ba–Zr film to prevent excessive oxidation of the precursors during the subsequent transfer and sulfurization. The SnS target, supplied by Pioneer Materials, Inc., was powered by pulsed DC for 15 min with a power density of 0.55 Wcm^–2^. The samples were rotated during all the depositions, which were carried out in Ar atmosphere with a source purity of 99.9997% at a working pressure of 0.57 Pa. All sputtering targets had a diameter of 7.62 cm (3 in.).

The as-sputter-deposited precursors were then sulfurized in a custom-built tube furnace with an approximate volume of 15 L and a base pressure of ca. 1 Pa. The setup was specifically designed for independent control of both the sample and the S source. The temperature inside the heating block, where the sample is normally placed during sulfurization, can be directly set. In contrast, the temperature of the source (*T*_S_) is determined by its distance from the heating block and is measured through a thermocouple connected to the S-containing vessel. A more detailed description of the furnace can be found in a previous work.^34^ To begin, the temperature of the heating block was set to the desired value and allowed to stabilize. Then, a load lock was vented with N, and the sample was placed on an open graphite holder. A separate small vessel was loaded with S powder provided by Thermo Scientific with a purity of 99.999%. The load lock was subsequently pumped to remove air traces before commencing sulfurization. The tube was then sealed and filled with Ar until a pressure of 400 Pa was reached. From this point onward, the sulfurization step followed two different procedures depending on the investigation type.

For the BaZrS_3_ Formation under Selected Conditions section, only the precursor was initially pushed toward the hot zone of the furnace—by means of a rod and a vacuum feedthrough—and allowed to heat. When *T*_sample_ reached 95% of the target value, the S-containing vessel was also slid toward the hot zone using a second rod and a second vacuum feedthrough. The sulfurization time was initiated once *T*_S_ reached 1 °C below the target. For the BaZrS_3_ Formation under Selected Conditions section, sulfurization times of 1, 2, and 5 min were used while ensuring that the S source was not depleted. This precaution was taken to prevent additional phase transformations (e.g., from BaS_3_ to BaS_2_) during the last phase of sulfurization due to a drop in *p*_S_. Both sample and S powder were finally gradually retracted into the load lock and allowed to cool to below 50 °C prior to unloading. It was assumed that S vapor was in equilibrium with its condensed phase during sulfurization, achieving a *p*_S_ corresponding to *T*_S_. For the conversion of one physical quantity to the other, the S vapor pressure curve from published experimental data was used,^35^ as detailed in a previous study.^34^ To minimize the contribution of background S, the furnace was always prebaked overnight before sulfurizing at low *T*_S_ (corresponding to BaS as the intermediate phase).

For the cross-experiments, instead, both sample and S source were moved simultaneously from the load lock toward the hot zone. When they reached the respective target temperatures, the timer for the sulfurization duration was started. The S-containing vessel was pulled away from the heating block after 10 min to allow for rapid cooling and to suppress further sulfurization. The sample was then either kept in the same position for an additional 10 min or moved to a higher target temperature for the same duration. At the end of the process, the sample was also retracted into the load lock and allowed to cool before being unloaded.

### Characterization

GI-XRD was performed using an Empyrean setup. The background noise was computationally subtracted from the raw diffraction pattern in HighScore Plus by PANalytical.^44^ The measured patterns were fitted using a Voigt function in combination with a Levenberg–Marquardt algorithm, from which FWHM and standard errors were calculated.

The cross-section preparation of different samples for the subsequent TEM analysis was conducted using a Focus Ion Beam (FIB) CrossBeam550 (ZEISS) following the standard lift-out procedure. To minimize beam-induced damage, the Ga-ion polishing conditions were gradually stepped down from 30 kV/300 pA to 2 kV/50 pA, rendering any potential damage negligible. The STEM data was acquired within a few days from the lamella preparation on a Cs-probe-corrected Titan Themis 200 (Thermofisher Scientific) operated at 200 kV.

## Conclusions

Ba–Zr precursors were sulfurized under varied conditions to evaluate the influence of different solid binary Ba sulfides as intermediates in the formation of BaZrS_3_ thin films. The processing temperature was deliberately maintained below the melting points of these phases to avoid the formation of any liquid flux. X-ray diffraction analysis indicates that the synthesized BaZrS_3_ films exhibit a higher degree of crystallinity when solid BaS_3_ serves as the intermediate phase, compared with BaS_2_, which in turn is more effective than BaS. Scanning transmission electron microscopy corroborates these findings, revealing that the BaZrS_3_ grain size increases with the order of the binary barium polysulfide involved. These observations suggest an enhancement of solid-state diffusion via an intercalation process of Zr into BaS_2_ and BaS_3_, analogous to the mechanisms reported in related (nonperovskite) materials.

To further disentangle the influence of sulfur partial pressure and sample temperature in BaZrS_3_ formation, these two parameters were methodically varied during the sulfurization process. X-ray analysis confirmed the superior role of BaS_3_ over BaS_2_. Furthermore, the key role of the trisulfide in initiating BaZrS_3_ nucleation is revealed, whereas an elevated sample temperature predominantly enhances the grain growth.

Through a systematic exploration of distinct synthetic routes involving different BaS_*x*_ intermediate phases, this study provides valuable insights into the chemical formation of BaZrS_3_ perovskites at moderate temperatures. These findings are also expected to be extended to other Ba-based multinary sulfides.

## Supplementary Material



## Notes

GI-XRD diffraction patterns of samples from Experiment B and Experiment C sulfurized for 1, 2, and 5 min (Figure S1) (PDF)
