# Supplementary material for: Synthesis of BaZrS3 Perovskite Thin Films via Different Solid BaS x Intermediate Phases
Source: ACS Omega. 2026 Jul 10;11(29):43412–8. doi: 10.1021/acsomega.6c01800 (PMC13425332; doi:10.1021/acsomega.6c01800)
Supplement: Supplementary file 1 [file ao6c01800_si_001.pdf]

Synthesis of $BaZrS_3$ Perovskite Thin Films via Different Solid BaS_x Intermediate Phases

SUPPORTING INFORMATION

Corrado Comparotto,[*a] Younes Lablali,[b] Olivier Donzel-Gargand,[a] Tomas Kubart,[c] Francesco Stancari,[d] and Jonathan J. S. Scragg[a]*

a. Division of Solar Cell Technology, Department of Materials Science and Engineering, Uppsala University, Uppsala 75103, Sweden. Email: corrado.comparotto@gmail.com

b. Department of Materials Science, Energy and Nano-engineering, Mohammed VI Polytechnic University, Benguerir 43150, Morocco

c. Division of Solid-State Electronics, Department of Electrical Engineering, Uppsala University, Uppsala 75103, Sweden

d. Department of Chemistry, Life Sciences and Environmental Sustainability, University of Parma, Parma 43124, Italy

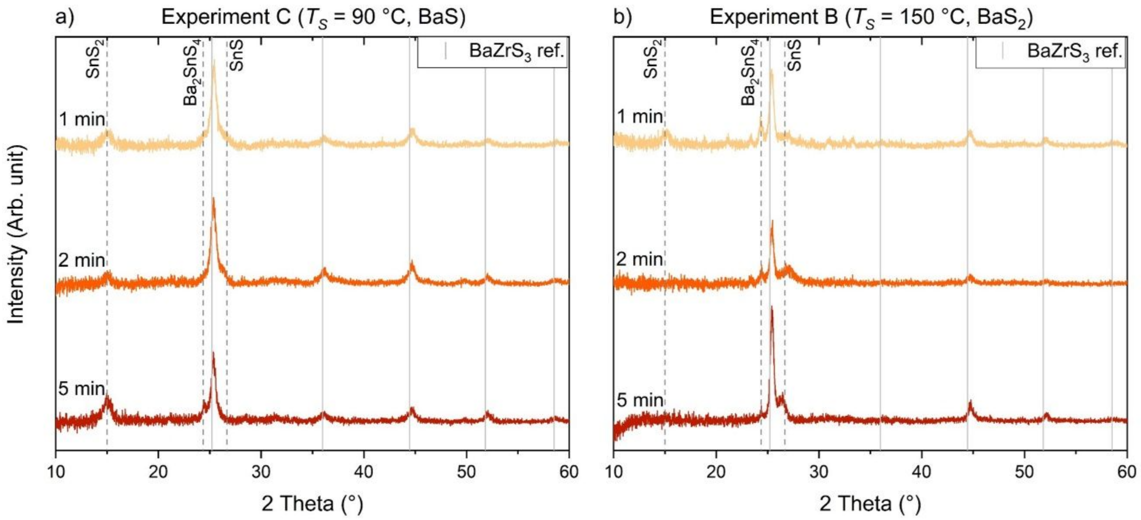

Figure S1: GI-XRD diffraction patterns of samples from a) Experiment C and b) Experiment B. The reference pattern for $BaZrS_3$, Ba_2SnS_4, SnS, and SnS_2 are taken from Lelieveld and Ijdo,[1] Susa and Steinflink,[2] Avellaneda et al.,[3] and Pałosz and Salje,[4] respectively.

REFERENCES

1 Lelieveld, R.; IJdo, D. J. W. Sulphides with the $GdFeO_3$ Structure. *Acta Crystallogr B Struct Sci* **1980**, *36* (10), 2223–2226. https://doi.org/10.1107/S056774088000845X.

2 Susa, K.; Steinfink, H. Ternary Sulfide Compounds AB_2S_4: The Crystal Structures of $GePb_2S_4$ and $SnBa_2S_4$. *Journal of Solid State Chemistry* **1971**, *3* (1), 75–82. https://doi.org/10.1016/0022-4596(71)90010-7.

3 Avellaneda, D.; Nair, M. T. S.; Nair, P. K. Polymorphic Tin Sulfide Thin Films of Zinc Blende and Orthorhombic Structures by Chemical Deposition. *J. Electrochem. Soc.* **2008**, *155* (7), D517. https://doi.org/10.1149/1.2917198.

4 Pałosz, B.; Salje, E. Lattice Parameters and Spontaneous Strain in AX_2 Polytypes: CdI_2, PbI_2, SnS_2, and $SnSe_2$. *J Appl Crystallogr* **1989**, *22* (6), 622–623. https://doi.org/10.1107/S0021889889006916.
